# The Prevention of Childhood Obesity Is a Priority: The Preliminary Results of the “EpPOI: Education to Prevent Childhood Obesity” Project

**DOI:** 10.3390/nu16152538

**Published:** 2024-08-02

**Authors:** Debora Porri, Giovanni Luppino, Letteria Anna Morabito, Elisa La Rosa, Giorgia Pepe, Domenico Corica, Mariella Valenzise, Maria Francesca Messina, Giuseppina Zirilli, Alessandra Li Pomi, Angela Alibrandi, Debora Di Mauro, Tommaso Aversa, Malgorzata Gabriela Wasniewska

**Affiliations:** 1 Department of Human Pathology of Adulthood and Childhood, University of Messina, Via Consolare Valeria, 98124 Messina, Italy; debora.porri@gmail.com (D.P.); giorgia.pepe@unime.it (G.P.); domenico.corica@unime.it (D.C.); mariella.valenzise@unime.it (M.V.); francesca.messina@unime.it (M.F.M.); giuseppina.zirilli@polime.it (G.Z.); alessandra.lipomi92@gmail.com (A.L.P.); tommaso.aversa@unime.it (T.A.); malgorzata.wasniewska@unime.it (M.G.W.); 2 Pediatric Unit, “G. Martino” University Hospital, 98124 Messina, Italy; letteria.morabito@gmail.com (L.A.M.); elisalarosa@icloud.com (E.L.R.); 3 Department of Economics, University of Messina, 98100 Messina, Italy; angela.alibrandi@unime.it; 4 Department of Biomedical and Dental Sciences and Morphological and Functional Imaging, University of Messina, 98100 Messina, Italy; debora.dimauro@unime.it

**Keywords:** childhood obesity, childhood obesity prevention, pediatric nutrition, lifestyle intervention

## Abstract

Background: The increase in childhood obesity rates represents a serious public health problem. The project “EpPOI: Education to prevent childhood obesity” is aimed at a multidisciplinary approach to raise awareness of the importance of preventing childhood obesity through lifestyle education. Methods: Two actions by experts were performed: an intervention with children in schools and a meeting for both parents and school staff. Participants completed a questionnaire structured as a Likert scale. Results: The sample size was 96 people, and awareness of the childhood obesity problem as well as the need for obesity prevention was high among respondents. We also found great interest among participants in having more information on pediatric nutrition and physical activity, with a positive correlation with age. Furthermore, the multivariate regression model configured interest in having more information on nutrition and physical activity as an independent and statistically significant predictor of awareness of childhood obesity as a current issue. Conclusions: The results highlight the need to act on childhood obesity through lifestyle prevention strategies early in life.

## 1. Introduction

Obesity is a global health problem and a growing public health concern, with an increasing incidence following the pediatric age [1]. It is already an epidemic problem. From 1975 to 2016, the prevalence of obesity in the age group of 5 to 19 years improved exponentially in both girls and boys, with a 10- and 12-fold increase, respectively [2]. The worldwide trend shows that about 41 million children under the age of 5 years are overweight, and 340 million children and adolescents aged 5 to 19 years are affected by obesity [2]. This growing trend concerns both developed countries and developing countries. In addition, the COVID-19 pandemic has globally amplified the spread of CO, and younger school-aged children and subjects with pre-pandemic conditions of overweight and obesity registered the largest weight gain [3]. The worrisome increase in CO rate is associated with a lowering of its onset age. Worldwide, about 40 million children under the age of 5 are affected by overweight or obesity, and in the United States, 1 in 8 preschoolers has obesity [4,5]. The prevalence rate of obesity is troubling in Europe and higher values of prevalence are registered in the Southern Europe and Mediterranean regions. In Italy, the prevalence of overweight and obesity in children aged 7–13 years reached 28.2% and 12.2%, respectively, in 2016 [6]. Italy is among the top countries with a high prevalence of overweight and obesity among 7- to 9-year-olds out of 36 European countries. Italy ranks fourth among 11-year-olds, and its ranking decreases with age [7]. In addition, the prevalence of overweight and obesity is higher in southern Italy than in northern Italy for both pediatric and adult populations [8].

Obesity is a chronic, multifactorial disease, and a wide range of determinants are implicated in its pathological mechanisms [1]. Sociocultural factors, ultra-processed foods, economics, and environmental determinants play a predominant role in childhood obesity [CO] unrelated to genetic causes. These factors have become predominant during the last few decades and contribute to negative lifestyle changes; therefore, the impact of cultural and socioeconomic environments on childhood and adolescent obesity is more decisive than other factors, such as genetic conditions [9]. The growing trend toward sedentariness as an obesogenic behavior is linked to excessive screen use, whether for games, social media use, or Internet surfing. In addition, time devoted to exercise in Italian schools is lower than in other European countries, and the recommended level of physical activity per day in Italy is among the lowest in Europe [10]. 

CO, primarily if severe and early-onset obesity, is linked to several complications, such as dyslipidemia, type 2 diabetes, and high blood pressure, that seriously increase the risk of cardiovascular diseases [11]. A strong association between body max index [BMI] and cardiovascular diseases is well known. Cardiovascular damage is more severe if BMI gain and metabolic–vascular alterations, such as hypercholesterolemia and endothelial cell dysfunction, occur from preschool age [12]. In addition, the risk of any cardiovascular event is positively associated with BMI values in children [13,14].

Another special feature of CO is the persistence of this condition during adolescence, which is a predictor of obesity in adulthood. In addition, around 80% of adolescents with obesity will still be obese in adulthood, and this trend is confirmed in subjects over age 30 [15]. Obesity is a predictor of lifetime morbidity, premature death, and adult mortality. Coronary heart disease and stroke are the leading causes of death in patients with a history of CO [16]. Another relevant aspect in the management of childhood obesity is the insufficient therapeutic response rate and high dropout rate during follow-up, mainly among adolescents and patients with no glucometabolic alterations [17].

Prevention interventions must be implemented early to counter the increasing incidence of CO, the high risk of chronic complications, and the potential mortality rate in this class of patients. Childhood is the period during which dietary and physical activity habits are established [18]. Therefore, lifestyle interventions aimed at correct nutrition and physical education should be actively promoted with strategies in school, preschool, community, and home settings. Several pieces of evidence suggest more benefits from school and preschool interventions, characterized by a process of education on adequate nutrition and physical activity, compared to community and home settings. In addition, dietary and physical activity interventions should be integrated with multicomponent interventions to improve the results of obesity prevention interventions for children aged 1–5 years [19].

The objective of the EpPoi project is to promote an innovative, multidisciplinary, and coordinated approach to prevent CO by raising awareness of the importance of healthy lifestyles, which include a balanced diet and adequate levels of physical activity, involving both parents and school staff. We presented the preliminary results regarding the degree of interest in nutrition and physical activity in childhood and the degree of awareness of CO and its health consequences. The degree of knowledge of health problems linked to obesity represents the first step toward improving the effectiveness of prevention methods, especially in childhood.

## 2. Materials and Methods

### 2.1. Objectives

**Figure 1:**
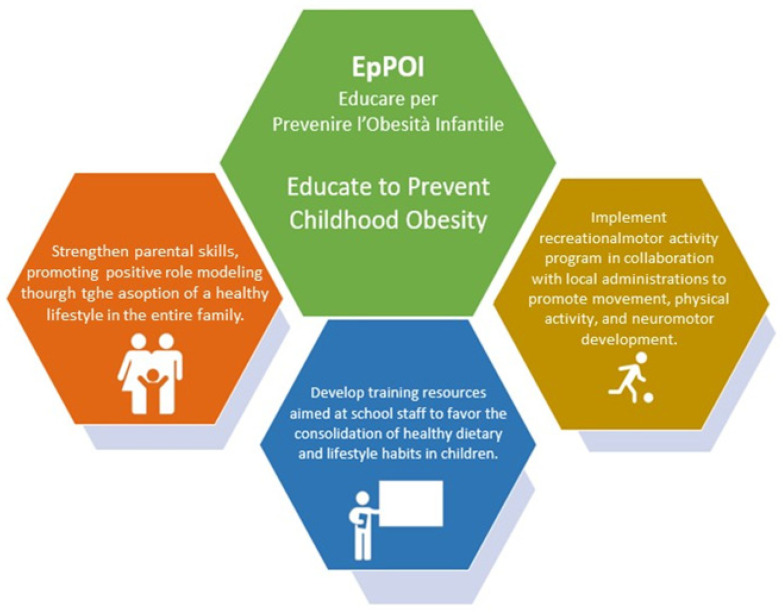
Milestones of EpPOI project.

The primary objective of the EpPoi project is to promote a multidisciplinary, intersectoral, and coordinated approach in order to raise awareness of the importance of early CO prevention through the promotion of a healthy lifestyle, including a balanced diet and adequate physical activity levels. To achieve this important purpose, some milestones are needed, highlighted in Figure 1.

The critical issues in which the EpPoi project is intended to intervene, and therefore the needs that are proposed to be satisfied as a result, are summarized below:

- Insufficient parental perception of body weight and habits of the child;
- An excess of sedentary lifestyle during childhood;
- Unhealthy eating habits during childhood;
- Structural aspects and the promotion of correct lifestyles at school;
- Scarce usability and traceability of information, the failure to involve all of the figures present in the life of children, and the sectorality and fragmentation of the interventions aimed at CO prevention.

The preliminary results of the EpPoi project were obtained with the first actions taken in nursery and nursery schools in order to gather critical issues and strengths to structure early and effective preventive interventions.

### 2.2. Selection Criteria

The target population was children from 0 to 6 years of age, of both sexes and any ethnicity, and their families. The study population also included school staff. The subjects were recruited directly from the school after an agreement with the school principal. School staff previously informed parents/caregivers and invited them to the meeting with the specialists. We included all children, parents, and school staff without any exclusion criteria.

### 2.3. Setting and Intervention

The first experimental approach was conducted by dividing participants into two groups based on age: 0–2 years old and 3–6 years old. The project was carried out at nursery Antonella Cocchiara for the 0–2 years group and at Ex csa 4^ Istituto Comprensivo Leopardi for the 3–6 years group, in Messina. 

We conducted an intervention that involved both training and practical approaches; weekly meetings were held for the 0–2 years age group, in which nursery staff and families were involved, introducing greater awareness of the role of motor skills from neonatal age and correct nutrition from the first years of life, including the importance of the first 1000 days, the mother’s choices during pregnancy, and the father role’s in his children’s health and development. 

As regards the 3–6 years age group, the project involves the school at three levels, information and practices directly for children, training meetings, and preventive education for parents and school staff. Meetings and interventions were performed by trained experts in the field of pediatric obesity, pediatricians, endocrinologists, nutritionists, kinesiologists, and psychologists. The experts clearly explained the seriousness and consequences of CO, teaching parents and school staff about the importance of adopting a healthy lifestyle early to ensure the health of their children. The meeting was an opportunity to discuss the principles of correct pediatric nutrition and the importance of adequate levels of physical activity structured according to age. Furthermore, through recreational activities, games, and dances aimed at nutritional and physical education, the experts also trained the children during dedicated school hours. Interventions, meetings, and data collection occurred between January and March 2024.

### 2.4. Outcome Measures

In order to evaluate parents and school staff’s involvement and interest, we performed an online survey by using a Likert scale and some demographic questions. The survey is made up of 6 questions in the form of a Likert scale, where 1 indicates “a little” and 5 indicates “a lot”, aimed at obtaining information about the degree of interest of the interviewees regarding nutrition and physical activity during pediatric age, the degree of awareness of the problem of CO, and the eventual request for additional information in this field.

The complete record of the questions proposed is listed in Table 1.

The survey was submitted to both parents and school staff after the meetings.

There were no direct benefits for the respondents from participating in this study. Participants were fully instructed about the study aim and were also informed that by agreeing to fill in the questionnaire, they confirmed their participation, automatically providing informed consent. The anonymous online survey was administered by means of Google Forms and the data were downloaded as a Microsoft Excel sheet (Office 2021).

### 2.5. Statistical Analysis

Categorical variables were expressed as absolute frequencies and percentages, with the numerical variables as mean ± standard deviation [SD].

The Kolmogorov–Smirnov test was applied in order to investigate the distribution of numerical variables; since all variables were not normally distributed, the nonparametric approach was adopted for data analysis.

The Spearman correlation test was applied in order to assess the interdependence between measured variables.

Subsequently, our attention was focused on the variable “*Do you think childhood obesity is a current problem*?” which was dichotomized; in particular, we assigned a value of 0 to scores between 1 and 3 [extremes included] and a value of 1 to scores greater than or equal to 4 [extremes included]. This dichotomous variable was considered the outcome of interest; therefore, a univariate and multivariate logistic regression model was estimated in order to identify the significant predictive factors of this outcome. The covariates inserted in the model were age, education level, and all questions rated by using the Likert scale. The results of the univariate and multivariate models were expressed as OR, 95% C.I, and *p*-value.

A radar chart was made to show the average satisfaction score of the respondents for each item [measured on a Likert scale] investigated through the formulation of questions.

A *p*-value lower than 0.05 was considered statistically significant. All statistical analyses were performed by using SPSS for Windows, version 22. 

## 3. Results

The total number of subjects who completed the survey was 96. The majority of respondents were over 40 years old [40.6%], and 50% of them had a high school diploma, while 19.8% had a middle school education and 30.2 percent had a degree. We did not gather information on gender and which parent filled out the questionnaire. 

It is important to note that almost half of those interviewed [46.95%] say they obtain information relating to their children’s lifestyle through specialists in the sector like pediatric endocrinologists, nutritionists, and kinesiologists.

**Figure 2:**
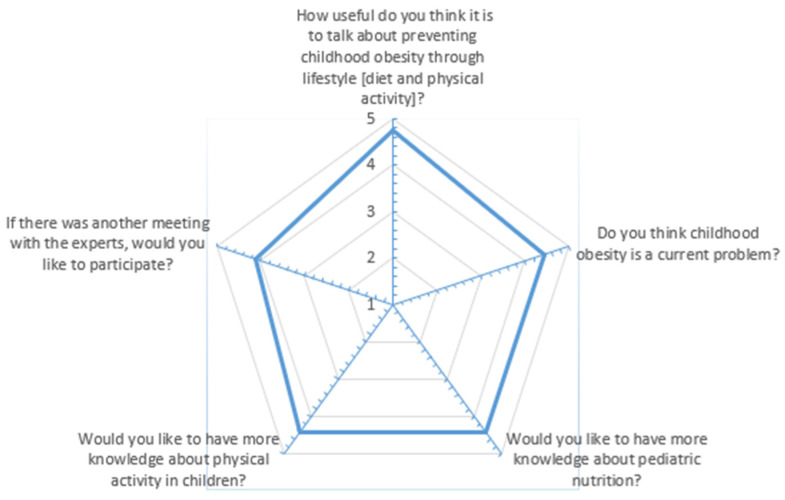
Radar chart.

The variables of our main interest are those investigated using the Likert scale, and the results are expressed as a radar chart (Figure 2).

The answers to the questions investigated by using the Likert scale are summarized in Table 2, expressed as absolute frequencies and percentages.

Interestingly, when asked “*Do you think childhood obesity is a current problem*?”, 63% assigned it a score of 5 on the Likert scale and only 16.7% assigned a value lower than 3. Over 70% of those interviewed consider the prevention of childhood obesity through lifestyle to be very useful, having assigned the question on this topic a score of 5 on the Likert scale. Furthermore, there was a high interest in having more information on pediatric nutrition and physical activity (respectively, 66.7% and 62.5% of interviewees assigned a score of 5 on the Likert scale).

We also assessed the interest in a new training meeting aimed at school staff and parents and a percentage of more than half of the sample, equal to 55.2%, declared a lot of interest in participating.

In order to perform a deep analysis, we evaluated the correlation between the variables of interest, as illustrated above (Table 2).

The first analysis included all of the variables of interest, i.e., the questions asked using the Likert scale, and we found a significant positive correlation between considering CO a current problem and considering the prevention of CO through lifestyle useful (*p* < 0.002). 

Furthermore, considering CO a problem correlates positively with interest in further knowledge regarding nutrition (*p* < 0.001) and physical activity in childhood (*p* < 0.001). We also found a significant correlation between interest in participating in a new meeting and considering CO a current problem (*p* > 0.001), recognizing the usefulness of CO prevention through lifestyle (*p* > 0.001), and interest in further knowledge regarding nutrition (*p* < 0.001) and physical activity in childhood (*p* < 0.001). 

All results are summarized in Table 3.

We also investigated the possible correlation between Likert-scale questions and some sample characteristics. 

Contrary to expectations, we did not find correlations with respondents’ education levels (Table 4), but with regard to age, we found a positive correlation between age and the question “*Do you think childhood obesity is a current problem?*” (*p* < 0.010) and the question “*Would you like to have more knowledge about pediatric nutrition?*” (*p* < 0.038) (Table 5).

We also employed a univariate and multivariate logistic regression model for our main outcome: “*Do you think childhood obesity is a current problem?*”.

The results are shown in Table 6.

The variables “Would you like to have more knowledge about pediatric nutrition” (*p* < 0.002, OR 2.31), “Would you like to have more knowledge about physical activity in children?” (*p* < 0.012, OR 2.00), and “If there was another meeting with the experts, would you like to participate?” (*p* < 0.022, OR 1.56) were positively correlated with the age of the respondents.

According to the multivariate regression model, variables “Would you like to have more knowledge about pediatric nutrition” and “Would you like to have more knowledge about physical activity in children?” were configured as independent and statistically significant predictors of the outcome of interest [“Do you think childhood obesity is a current problem?”] (respectively, *p* < 0.007, OR 2.09; *p* < 0.012, OR 2.0).

## 4. Discussion

CO rates are increasing worldwide, representing a current public health problem, especially due to the possibility of the disease extending into adulthood, with numerous consequences both on health and well-being and on healthcare costs [20,21,22]. 

The need for early intervention is based on the high risk of worsening excess weight by about 5-fold when an overweight condition is already present at 5 years of age. Indeed, once excess weight is established, the possibility of reversing it is very difficult, so the likelihood of becoming obese in adulthood increases [23].

Recent works have confirmed that certain periods of childhood are characterized by significant changes in adiposity, so they represent a valuable window of intervention for obesity prevention. These periods include the first 2 years of life, the period of the so-called “adiposity rebound” (between 5 and 7 years of age), and puberty [24,25].

Most of these assumptions inspired our “EpPOI project”, which provides an innovative CO prevention program in the early stages of life, involving parents and school staff, based on principles of a correct lifestyle with adequate nutrition and physical activity levels.

In the last few years, several CO prevention programs have been put in place, but only a few of them involved preschool children. A recent systematic review [26] examines the possibility of school-based interventions for promoting physical activities to prevent CO in primary and secondary school, highlighting a very high potential in reducing or preventing weight gain. Our results are in line with this hypothesis, considering both the high interest of survey participants in having more knowledge about physical activity in children and this interest as an independent and statistical predictor of considering CO a problem. 

It is well known that parental/caregiver awareness and involvement are crucial points of intervention in any pediatric disease primary prevention strategy, and this is also true for CO.

Several studies have shown that parents, particularly mothers, often underestimate their children’s weight, and the consequences of this misperception lead to less use of specific health services and interfere with the effectiveness of prevention programs [27,28].

The preliminary results of our survey are partially in line with these observations. We found a significant positive correlation between considering CO a current problem and considering prevention through lifestyle useful; in addition, participants’ awareness of CO as a current problem was high, as evidenced by the responses to the question “*Do you think childhood obesity is a current problem?*”.

Interestingly, the perception of CO as an emerging problem and the need for action increase with the age of respondents, as shown by the positive correlation observed between age and the two questions “*Do you think childhood obesity is a current problem*?” and “*Would you like to have more knowledge about pediatric nutrition?*”. To the best of our knowledge, parental age is not a highly considered factor in assessing the risk of childhood obesity, unlike other aspects such as parental weight status and socioeconomic and education level [29,30].

Contrary to our expectations, considering most of the evidence in the literature, we did not find a correlation between parents’ education level and their perception of CO as a problem. 

The results are promising and these preliminary observations reinforce the need to shift from the traditional paradigm of treating childhood obesity and its comorbidities to implementing CO prevention strategies that focus on preventing the development of overweight in early childhood.

Another relevant innovation of our EpPOI program is the multi-address approach based on the involvement of all of the people involved in the care of children, teachers, parents, and school staff, which would enable the extension of the correct prevention measures to all areas of a child’s life.

The main limitation of this study was the short time interval of the application of prevention measures, as it was a pilot study. Ideally, the duration and frequency of intervention sessions should be increased to improve their effectiveness. However, low parental adherence to the intervention could compromise its effectiveness. 

## 5. Conclusions

To sum up, our results highlight the need to act on CO through lifestyle prevention strategies early in life and to pursue the objectives of the EpPOI project, not only through actions aimed at children’s lifestyle changes but also through parents’ perception, correct lifestyle promotion at school, and the implementation of today’s poor usability and traceability of information concerning CO.

## Figures and Tables

**Table 1:** Complete questionnaire.

Question	Answer
1. Age group	>20
	20–25
	25–30
	30–35
	35–40
	>40
2. Education level	primary school diploma
	middle school diploma
	diploma
	degree
3. Where do you get information about nutrition and physical activity?	pediatricians
	internet
	family or friends
	specialists
4. How useful do you think it is to talk about preventing childhood obesity through lifestyle [diet and physical activity]?	from 1 [a little] to 5 [a lot]
5. Do you think childhood obesity is a current problem?	from 1 [a little] to 5 [a lot]
6. If there was another meeting with the experts, would you like to participate?	from 1 [a little] to 5 [a lot]
7. Would you like to have more knowledge about pediatric nutrition?	from 1 [a little] to 5 [a lot]
8. Would you like to have more knowledge about physical activity in children?	from 1 [a little] to 5 [a lot]
9. Have you made any changes in your teaching activity after the meeting with the specialists? [only for school staff]	from 1 [a little] to 5 [a lot]
9. Have you made any changes to your family lifestyle after meeting with the specialists? [only for parents]	from 1 [a little] to 5 [a lot]
10. What changes did you make?	

**Table 2:** Questions investigated by Likert scale as absolute frequencies and percentages.

Likert Scale [*n*]	Frequency [*n*]	Percentage [%]
4. How useful do you think it is to talk about preventing childhood obesity through lifestyle [diet and physical activity]?		
1	0	0
2	0	0
3	6	6.3%
4	14	76%
5	76	79.2%
5. Do you think childhood obesity is a current problem?		
1	1	1%
2	0	0
3	16	16.7%
4	18	18.8%
5	61	63.5%
6. If there was another meeting with the experts, would you like to participate?		
1	7	7.3%
2	4	4.2%
3	13	13.5%
4	19	19.8%
5	53	55.2%
7. Would you like to have more knowledge about pediatric nutrition?		
1	1	1%
2	4	4.2%
3	12	12.5%
4	15	15.6%
5	64	66.7%
8. Would you like to have more knowledge about physical activity in children?		
2	4	4.2%
3	14	14.6%
4	18	18.8%
5	60	62.5%

**Table 3:** Correlations between questions asked using Likert scale. Spearman correlation test, significance level *p* < 0.005. Significant correlations were highlighted as * (if *p*-value < 0.05), and ** (if *p*-value < 0.01).

		4. How useful do you think it is to talk about preventing childhood obesity through lifestyle [diet and physical activity]?	5. Do you think childhood obesity is a current problem?	6. If there was another meeting with the experts, would you like to participate?	7. Would you like to have more knowledge about pediatric nutrition?	8. Would you like to have more knowledge about physical activity in children?
4. How useful do you think it is to talk about preventing childhood obesity through lifestyle [diet and physical activity]?	Correlation coefficient	1.000	0.310 **	0.532 **	0.533 **	0.349 **
	*p*-value		0.002 *	0.000 *	0.00 *	0.00 *
5. Do you think childhood obesity is a current problem?	Correlation coefficient	0.310 **	1.000	0.430 **	0.348	0.329
	*p*-value	0.002 *		0.000 *	0.001 *	0.001 *
6. If there was another meeting with the experts, would you like to participate?	Correlation coefficient	0.532 **	0.430 **	1.000	0.770 **	0.531 **
	*p*-value	0.000 *	0.000 *		0.000 *	0.000 *
7. Would you like to have more knowledge about pediatric nutrition?	Correlation coefficient	0.553 **	0.348 **	0.770 **	1.000	0.571 **
	*p*-value	0.000 *	0.001 *	0.000 *		0.000 *
8. Would you like to have more knowledge about physical activity in children?	Correlation coefficient	0.349 **	0.329 **	0.531 **	0.571 **	1.000
	*p*-value	0.000 *	0.001 *	0.000 *	0.000 *	

**Table 4:** Correlations between questions asked using Likert scale and education level. Spearman correlation test, significance level *p* < 0.005.

		**Education Level**
Education level	Correlation coefficient	1.000
	*p*-value	
4. How useful do you think it is to talk about preventing childhood obesity through lifestyle [diet and physical activity]?	Correlation coefficient	0.13
	*p*-value	0.902
5. Do you think childhood obesity is a current problem?	Correlation coefficient	0.127
	*p*-value	0.218
6. If there was another meeting with the experts, would you like to participate?	Correlation coefficient	−0.051
	*p*-value	0.624
7. Would you like to have more knowledge about pediatric nutrition?	Correlation coefficient	0.089
	*p*-value	0.387
8. Would you like to have more knowledge about physical activity in children?	Correlation coefficient	0.96
	*p*-value	0.352

**Table 5:** Correlations between questions asked using Likert scale and age. Spearman correlation test, significance level *p* < 0.005. Significant correlations were highlighted as * (if *p*-value < 0.05), and ** (if *p*-value < 0.01).

		**Age**
Age	Correlation coefficient	1.000
	*p*-value	
4. How useful do you think it is to talk about preventing childhood obesity through lifestyle (diet and physical activity)?	Correlation coefficient	0.038
	*p*-value	0.712
5. Do you think childhood obesity is a current problem?	Correlation coefficient	0.263 **
	*p*-value	0.010 **
6. If there was another meeting with the experts, would you like to participate?	Correlation coefficient	0.131
	*p*-value	0.204
7. Would you like to have more knowledge about pediatric nutrition?	Correlation coefficient	0.212 *
	*p*-value	0.038 *
8. Would you like to have more knowledge about physical activity in children?	Correlation coefficient	1.66
	*p*-value	0.106

**Table 6:** Results of univariate and multivariate logistic regression model for outcome of interest [*Do you think childhood obesity is a current problem?*]. Significance level *p* < 0.005. Significant correlations were highlighted as * (if *p*-value < 0.05).

Variables	Univariate			Multivariate		
	OR	95% CI	*p*-Value	OR	95% CI	*p*-Value
Age	1.68	1.078–2.643	0.022 *	1.520	0.943–2.451	0.086
Education level	1.499	0.705–3.188	0.293	1.264	0.531–3.006	0.596
4. How useful do you think it is to talk about preventing childhood obesity through lifestyle [diet and physical activity]?	1.584	0.702–3.573	0.267	0.916	0.350–2.396	0.857
6. If there was another meeting with the experts, would you like to participate?	1.568	1.066–2.307	0.022 *	1.170	0.709–1.929	0.539
7. Would you like to have more knowledge about pediatric nutrition?	2.315	1.373–3.903	0.002 *	2.093	1.222–3.584	0.007 *
8. Would you like to have more knowledge about physical activity in children?	2.001	1.162–3.445	0.012 *	2.001	1.162–3.445	0.012 *

## Data Availability

Data is contained within the article.
